# l‐arginine ingestion inhibits eccentric contraction‐induced proteolysis and force deficit via *S*‐nitrosylation of calpain

**DOI:** 10.14814/phy2.13582

**Published:** 2018-01-25

**Authors:** Keita Kanzaki, Daiki Watanabe, Chihiro Aibara, Yuki Kawakami, Takashi Yamada, Yoshitaka Takahashi, Masanobu Wada

**Affiliations:** ^1^ Department of Clinical Nutrition Faculty of Health Science and Technology Kawasaki University of Medical Welfare Okayama Japan; ^2^ Graduate School of Integrated Arts and Sciences Hiroshima University Hiroshima Japan; ^3^ Department of Nutritional Science Faculty of Health and Welfare Science Okayama Prefectural University Okayama Japan; ^4^ Graduate School of Health Sciences Sapporo Medical University Hokkaido Japan

**Keywords:** Ca^2+^ regulatory proteins, nitric oxide, NO synthase, sarcoplasmic reticulum

## Abstract

It has been shown that calpains are involved in the proteolysis of muscle proteins that occurs with eccentric contraction (ECC) and that exogenously applied nitric oxide decreases the calpain‐mediated proteolysis. The aim of this study was to examine the effects of ingestion of l‐arginine (ARG), a nitric oxide precursor, on ECC‐related calpain activation. In the first and second experiments, male Wistar rats were given ARG in water for 7 days starting from 3 days before the ECC protocol (average ingestion, ~600 mg kg‐body wt^−1^ day^−1^). Tibialis anterior muscles underwent 200 repeated ECCs and, subsequently, were excised 3 days later. Whole muscle analyses (the first experiment) revealed that ARG attenuated ECC‐induced force deficit and autolysis of calpain‐1, and increased the amounts of *S*‐nitrosylated calpain‐1. Regarding ryanodine receptor (RyR) and dihydropyridine receptor (DHPR), ECC‐induced proteolysis was completely inhibited by ARG, whereas the inhibition was partial for junctophilin‐1 (JP1). Skinned fiber analyses (the second experiment) showed that ARG also inhibited ECC‐elicited reductions in the ratio of depolarization‐induced to maximum Ca^2+^‐activated force. In the third experiment, homogenates of rested muscles were treated with *S*‐nitrosylating agent, *S*‐nitrosoglutathione (GSNO), and/or high Ca^2+^ concentration ([Ca^2+^]). Treatment with high [Ca^2+^] and without GSNO produced proteolysis of RyR, DHPR, and JP1. On the other hand, treatment with high [Ca^2+^] and GSNO caused complete inhibition of RyR and DHPR proteolysis and partial inhibition of JP1 proteolysis. These results indicate that ARG ingestion can attenuate ECC‐induced proteolysis of Ca^2+^ regulatory proteins and force deficit by decreasing calpain activation via *S*‐nitrosylation.

## Introduction

Eccentric contraction (ECC) is a contraction in which skeletal muscles are stretched while contracting. It is a part of normal activities such as walking downstairs or lowing a heavy weight, and is prone to result in larger and longer‐lasting force deficits compared with concentric and isometric contractions (Lavender and Nosaka 2006; Kanzaki et al. 2010). This is primarily because of muscle damage characterized by triad deformation (Takekura et al. 2001), sarcomere inhomogeneity (Balnave et al. 1997), increased membrane permeability (Lavender and Nosaka 2006), inflammation (Liao et al. 2010), and proteolysis (Kanzaki et al. 2010, 2014; Zhang et al. 2012).

Skeletal muscles are endowed with three proteolytic systems: autophagy–lysosomal proteases, ATP‐dependent ubiquitin‐related proteasome complex, and Ca^2+^‐regulated cysteine (Cys) proteases (calpains). In a recent study from our laboratory, it was shown in fast‐twitch muscles of rats that treatment with a calpain inhibitor can attenuate ECC‐elicited force deficits and proteolysis of junctophilin (JP) and dihydropyridine receptor (DHPR) that regulate Ca^2+^ release from the sarcoplasmic reticulum (SR; Kanzaki et al. 2017). Our observations suggest that: (1) impaired function of these proteins is responsible, at least in part, for ECC‐dependent force deficits; (2) calpains are primarily involved in proteolysis with ECC; and (3) inhibition of calpain activation is one of the effective strategies in overcoming reduced muscle performance associated with ECC.

In skeletal muscles, nitric oxide (NO), which is synthesized from l‐arginine (ARG) primarily by neuronal NO synthase (NOS), is moderately generated in the resting state, and its production increases markedly with contractile activity (Stamler and Meissner 2001; Wray et al. 2011; Hong et al. 2014; Dyakova et al. 2015). Modest and transient increases in NO play essential roles in the normal cellular signaling (McConell et al. 2012). On the other hand, large or prolonged levels of NO result in impaired function of the contractile apparatus via the formation of *S*‐nitrosylation and/or peroxynitrite (Dutka et al. 2011a, 2017). Previous in vitro studies on the effect of NO on calpains have demonstrated that the use of NO donors can inhibit calpain activation, which is mediated through *S*‐nitrosylation (Michetti et al. 1995; Liu et al. 2016). Considering these findings and the fact that calpain‐related proteolysis with aging is ascribable to a loss of NOS (Samengo et al. 2012), it seems quite plausible that NO would perform a role in protecting muscle proteins from calpain‐induced proteolysis in vivo.

Recently, Lomonosova et al. (2014) reported that in rats, ingestion of ARG before a single bout of ECC resulted in increased NO production in quiescent slow‐twitch muscles, decreased calpain‐1 mRNA up‐regulation and desmin degradation in contracted slow‐twitch muscles, and improved the running performance a few days after ECC, compared to controls. Their observations, together with previous findings from in vitro studies (Michetti et al. 1995; Liu et al. 2016), suggest that in skeletal muscles, ARG may induce reduced enzymatic activation of calpain‐1 via the down‐regulation and/or *S*‐nitrosylation of the enzyme, resulting in reduction of the muscle protein degradation and force deficits that occur with ECC. However, in this regard, some considerations arise in light of the following previous findings: (1) all studies, except for one by Lomonosova et al. (2014), showed no changes in calpain‐1 mRNA levels or protein expression in muscles subjected to ECC (Feasson et al. 2002; Serra et al. 2012; Kanzaki et al. 2014); (2) JP, DHPR, and ryanodine receptor (RyR), all of which play a critical role in the regulation of [Ca^2+^]_i_, are less susceptible to calpain‐elicited proteolysis, compared to force‐transmitting proteins (e.g., desmin, titin, and dystrophin; Lieber et al. 1996; Kanzaki et al. 2017).

In this study, two questions were investigated as to whether ARG ingestion prior to ECC is capable of mitigating: (1) ECC‐induced calpain activation via *S*‐nitrosylation of the enzyme; and (2) ECC‐dependent proteolysis of Ca^2+^ regulatory proteins. Furthermore, the effects of ARG ingestion and ECC on the SR Ca^2+^ release and myofibril functions were examined using mechanically skinned fibers. The experiments conducted with fast‐twitch muscles of rats reveal that ARG ingestion can inhibit ECC‐induced proteolysis of Ca^2+^ regulatory proteins and force deficit by decreasing the proteolytic function of calpains, which result from *S*‐nitrosylation of the enzyme.

## Methods

### Ethical approval

All experimental procedures performed in this study were approved by the Animal Experimental Committee of Okayama Prefectural University and Hiroshima University. Animal care was in accordance with the institutional guidelines, which are based on the Act on Welfare and Management of Animals, the Standards Relating to the Care and Management of Laboratory Animals and Relief of Pain, the Fundamental Guidelines for Proper Conduct of Animal Experiment and Related Activities in Academic Research Institutions, and other related laws and regulations in Japan. All experiments complied with the policies of *The Journal of Physiology* written by Drummond (2009).

### Outline of study and animal care

In this study, we performed three separate experiments (1–3) with the following aims: (1) whether ARG ingestion facilitates recovery of force production after ECC and whether the recovery is because of decreased proteolysis; (2) whether ARG ingestion attenuates ECC‐induced disturbances in SR Ca^2+^ release and myofibril function; (3) whether in vitro *S*‐nitrosylation of calpain decreases Ca^2+^‐elicited proteolysis, respectively.

The experiments were performed in male Wistar rats (*n* = 29; Charles River Laboratories, Yokohama, Japan). They were provided standard chow (CE‐2; CLEA Japan, Tokyo, Japan) containing 1.53% (mass mass^−1^) ARG and water ad libitum, and were individually housed in a cage in a thermally controlled room (~24°C) with 12‐h light/dark cycle. An intraperitoneal injection of a mixture of medetomidine (0.4 mg kg‐body wt^−1^), midazolam (2.0 mg kg‐body wt^−1^), and butorphanol (2.5 mg kg‐body wt^−1^) was used for anesthesia. At the end of experiments, the rats were euthanized with pentobarbital sodium (200 mg kg‐body wt^−1^), followed by cervical dislocation.

### Experiment 1

#### ARG ingestion and ECC protocol

Rats aged 8–9 weeks were randomly assigned to a control (CON) or an ARG group (*n* = 7 for each group). ARG rats were provided water containing 0.3% (mass vol.^−1^) ARG (Wako Pure Chemical, Osaka, Japan) ad libitum for 7 days starting from 3 days before the ECC protocol and continuing for 3 days after ECC (average ingestion, 611 mg kg‐body wt^−1^ day^−1^). On the contrary, age‐matched controls were given water without ARG. Based on the chow intake (24.7 g day^−1^) and the ARG content (see above) in the chow, on the average, rats were estimated to ingest ~990 mg kg‐body wt^−1^ day^−1^ of ARG from food. The supplemental dose (611 mg kg‐body wt^−1^ day^−1^) of ARG used in this study is similar to that utilized in the study by Lomonosova et al. (2014) and equivalent to 98 mg kg‐body wt^−1^ day^−1^ in humans (Yang et al. 2015). Such an ARG dose has frequently been used in human studies (Bescos et al. 2012) and has been confirmed to be safe for both humans and rats (Yang et al. 2015; Cynober et al. 2016).

Eccentric contraction was performed as described previously (Kanzaki et al. 2017). Briefly, under anesthesia, ECCs were elicited in the anterior crural muscles by stimulating the left peroneal nerve using a 1‐sec train of a 1‐msec pulse at 50 Hz and supramaximal voltage during forced plantar flexion (150° angular movement at 150° sec^−1^). ECCs were repeated every 4 sec for 200 cycles.

#### In situ and in vitro measurements of isometric force

Three days after ECC, isometric force was measured in situ and in vitro. This time point was selected because ECC results in force deficits and proteolysis of Ca^2+^ regulatory proteins at this time (Kanzaki et al. 2014, 2017). The rats were placed in a supine position under anesthesia and their limbs were attached to a foot holder connected to an isometric transducer (TB‐611T; Nihon Koden, Tokyo, Japan). Isometric contractions of the left (ECC imposed) and right (rested) anterior crural muscles were elicited by stimulating the peroneal nerve with a 1‐sec train of a 1‐msec pulse at various frequencies (1–80 Hz; in situ measurement).

Following the in situ measurements, extensor digitorum longus (EDL) muscles were excised from both the legs. The muscles were mounted in a chamber (30°C) between a force transducer and an adjustable holder, and were perfused with standard Krebs–Ringer solution consisting of (in mmol L^−1^): 115 NaCl, 20 NaHCO_3_, 11 glucose, 5 KHCO_3_, 5 *N,N*‐bis(2‐hydroxyethyl)‐2‐aminoethanesulfonic acid, 2 CaCl_2_, 1 MgCl_2_, 0.38 glutamine, and 0.3 glutamic acid. The solution was continuously bubbled with 95% O_2_–5% CO_2_, giving a bath pH of 7.4. Isometric contractions were elicited by direct stimulation via two platinum plate electrodes with a 1‐sec train of a 1‐msec pulse at various frequencies (1–80 Hz; in vitro measurement). The resulting forces were recorded on a personal computer and analyzed using LabChart version 8. After the in vitro measurements, tibialis anterior (TA) muscles were excised from both legs, frozen in liquid nitrogen, and stored at −80°C for biochemical analyses to be performed later. EDL muscles were not used for biochemical analyses because of restrictions on the amount of this muscle available.

#### Immunoblot

Immunoblot analyses were performed as described previously (Kanzaki et al. 2017). TA muscles (~50 mg) were homogenized in 9 vol. (vol. mass^−1^) of an ice‐cold buffer consisting of 20 mmol L^−1^ Tris (pH 7.4), 5 mmol L^−1^ EDTA, 5 mmol L^−1^ EGTA, 1 mmol L^−1^ dithiothreitol, 0.5 mmol L^−1^ phenylmethanesulphonyl fluoride, 14 *μ*mol L^−1^ pepstatin A, and 10 *μ*g mL^−1^ 4‐(2‐aminoethyl)‐benzenesulfonylfluoride. The protein content of the homogenate was determined by the Bradford assay using bovine serum albumin as the standard (Bradford 1976). Proteins (8 *μ*g) were electrophoretically separated and transferred to polyvinylidene difluoride membranes. The membranes were incubated overnight at 4°C with primary antibodies as follows: anti‐RyR1/2 (1:2500 dilution; MA‐925; Thermo Fisher Scientific, Waltham, MA), anti‐DHPR *α*1 subunit (1:1000 dilution; MA3‐920; Thermo Fisher Scientific), anti‐JP1 (1:1000 dilution; 40‐5100; Thermo Fisher Scientific), anti‐calsequestrin (CSQ) 1 (1:1000 dilution; sc‐53012; Santa Cruz Biotechnology, Dallas, TX), and anti‐calpain‐1 (1:1000 dilution; C0355; Sigma, St. Louis, MO). The membranes were then incubated with secondary antibodies (1:5000 dilution, sc‐2004 for JP1, Santa Cruz Biotechnology; 1:5000 dilution, P0260 for DHPR, RyR, CSQ1, and calpain‐1, Agilent Technologies, Santa Clara, CA) for 1 h at room temperature. To quantify the total proteins on the membrane, it was stained with Rapid Stain Coomassie blue R Kit (30035‐14, Nacalai Tesque, Kyoto, Japan). The contents of four proteins investigated (RyR, DHPR, JP1, and CSQ1) were normalized to total proteins.

#### Nitrite/nitrate concentration measurements

In a preliminary experiment, we observed that ECC evoked increases in the amounts of muscle nitrite and nitrate (NOx) and that the increases were suppressed by an oral ingestion (60 mg kg‐body wt^−1^ day^−1^) of a NOS inhibitor, *N*(G)‐nitro‐l‐arginine‐methyl ester, indicating that NOx levels can be used as an indicator of the amounts of endogenously produced NO. NOx levels were spectrophotometrically determined as described previously (Miranda et al. 2001). TA muscles (~50 mg) were pulverized under liquid nitrogen and vortexed with 6 vol. (vol. mass^−1^) of ice‐cold PBS (pH 7.4) containing 10 mmol L^−1^ *N*‐ethylmaleimide and 2.5 mmol L^−1^ EDTA. The mixture was then centrifuged at 10,000 *g* for 5 min at 4°C and the resultant supernatant was collected. After adding equal amounts of acetonitrile, the mixture was vortexed and centrifuged as mentioned above. The supernatant (100 *μ*L) was mixed with equal amounts of 8 mg mL^−1^ vanadium (III) chloride, followed by Griess reagents (100 *μ*L) consisting of 1% (mass vol.^−1^) sulfanilamide and 0.05% (mass vol.^−1^) *N*‐(1‐naphthyl) ethylendiamine dihydrochloride. In the experiments, the Griess reagent in the controls was replaced with 2.5% (vol. vol.^−1^) HCl. After incubation at 37°C for 30 min, the absorbance was read at 540 nm.

#### Resin‐assisted capture of *S*‐nitrosothiol (SNO‐RAC)

To evaluate the contents of *S*‐nitrosylated calpain‐1, the SNO‐RAC technique was utilized as described previously (Guo et al. 2014; Figueiredo‐Freitas et al. 2015). The assay specificity of SNO‐RAC for *S*‐nitrosylated Cys residues was ascertained in experiment 3. The SNO‐RAC procedures were conducted as follows.

##### Step 1 (sample preparation)

Tibialis anterior muscles (~50 mg) were homogenized in 5 vol. (mass vol.^−1^) of ice‐cold RIPA buffer consisting of 150 mmol L^−1^ NaCl, 50 mmol L^−1^ Tris/HCl (pH 7.4), 5 mmol L^−1^ NaF, 1 mmol L^−1^ NaVO_4_, and 1% (vol. vol.^−1^) Triton X‐100. The homogenate was centrifuged at 10,000 *g* for 5 min at 4°C and the resultant supernatant was collected.

##### Step 2 (blockage of free thiols)

To block free thiols, the supernatant was incubated with a solution composed of 0.1% (vol. vol.^−1^) *S*‐methyl methanethiosulfonate and 2.5% (mass vol.^−1^) SDS at 50°C for 20 min under agitation. After the addition of 3 vol. of acetone, the mixture was allowed to precipitate for 20 min at −20°C, followed by centrifugation at 3000 *g* for 10 min at 4°C. The pellet was washed twice in 70% acetone to remove all excess *S*‐methyl methanethiosulfonate and was resuspended in PBS containing 1 mmol L^−1^ EDTA and 0.1 mmol L^−1^ neocuproine (PEN buffer). The protein concentrations in the samples were adjusted with the PEN buffer to achieve equal concentrations (1 mg mL^−1^) and a small fraction (10 *μ*L) of each sample was removed for analysis of protein “input” to SNO‐RAC.

##### Step 3 (reduction of *S*‐nitrosylated thiols and protein capture)

After *S*‐nitrosylated thiols were reduced to free thiols by adding 5 *μ*mol L^−1^ CuCl and 5 mmol L^−1^ sodium ascorbate (Cu‐Asc treatment), to capture proteins containing free thiols, the samples were incubated with the resin slurry containing 15 mg thiopropyl sepharose 6B for 2 h at room temperature.

##### Step 4 (protein elution)

Resins were washed in the PEN buffer containing 1% (mass vol.^−1^) SDS (PENS solution) and were centrifuged at 1000 *g* for 10 sec at room temperature. This procedure (wash and centrifugation) was repeated five times. The proteins captured were eluted by incubating the resins with PENS solution containing 100 mmol L^−1^ *β*‐mercaptoethanol for 20 min. Following centrifugation at 1000 *g* for 10 sec, the supernatant was collected. Steps 2–4 were performed in the dark to avoid light‐induced reduction of ascorbate and photolysis of *S*‐nitrosylated sites.

##### Step 5 (detection of calpain‐1)

Calpain‐1 was detected using the immunoblot procedure as described above and the contents were evaluated relative to the band density of “input”.

### Experiment 2

#### ARG ingestion and ECC protocol

In experiment 2, skinned fiber experiments were performed. In the preliminary experiments, when muscles from rats weighing <450 g were used, it was not easy to prepare skinned fibers with their excitation–contraction coupling mechanism intact. For this reason, rats aged 12–13 weeks (average body wt, 500 g) were used in the experiment 2 (*n* = 5 for both CON and ARG groups). The procedures for ARG ingestion and ECC were similar to those used in experiment 1, with the exception of the ARG concentration (0.6%) in drinking water (average ingestion of ARG, 605 mg kg‐body wt^−1^ day^−1^). This is because relative water intake (per body weight) declines as body weight increases. On an average, rats were estimated to ingest ~720 mg kg‐body wt^−1^ day^−1^ of ARG from food.

#### Skinned fiber preparation

Three days after ECC, TA muscles were excised from both legs and mechanically skinned fibers were prepared as described previously (Watanabe et al. 2015; Watanabe and Wada 2016). One‐ to three‐skinned fibers were obtained from one whole muscle. A segment of the skinned fiber was connected to a force transducer (muscle tester, SI, Germany) and transferred to a bath containing 2 mL of K‐hexamethylethylene‐diaminetetraacetic acid (HDTA) solution (see solution). All skinned fibers (*n* = 41) were initially subjected to measurements of depolarization (depol)‐induced force responses, followed by caffeine‐induced and maximum Ca^2+^ (max Ca^2+^)‐activated force responses. Analyses of force–Ca^2+^ relationship were also done in some skinned fibers (*n* = 28).

#### Solution

All solutions for skinned fiber experiments were prepared as described previously (Watanabe et al. 2015; Watanabe and Wada 2016). The solutions were composed of 36 mmol L^−1^ Na^+^, 126 mmol L^−1^ K^+^, 90 mmol L^−1^ Hepes, 8 mmol L^−1^ ATP_total_, and 10 mmol L^−1^ creatine phosphate, and had a pH of 7.09–7.11 at 26°C. The K‐HDTA solution additionally contained 0.05 mmol L^−1^ EGTA and 50 mmol L^−1^ HDTA to give 10^−6.9^ mol L^−1^ free [Ca^2+^]. Na‐HDTA solution was similar to the K‐HDTA solution, with K^+^ replaced with Na^+^. Free [Mg^2+^] was set at 1.0 mmol L^−1^ except in the Ca^2+^ release solution, which was similar to the K‐HDTA solution but with 0.05 mmol L^−1^ free [Mg^2+^], 30 mmol L^−1^ caffeine, and 0.5 mmol L^−1^ EGTA. The max Ca^2+^ solution was also similar to the K‐HDTA solution, but with HDTA replaced with 49.5 mmol L^−1^ Ca‐EGTA and 0.5 mmol L^−1^ free EGTA, whereas the relaxation solution contained 50 mmol L^−1^ free EGTA. These two solutions were mixed in an appropriate ratio with free [Ca^2+^] in the range of 10^−9^–10^−4.7^ mol L^−1^, and used for force–[Ca^2+^] relationship analysis.

#### Na^+^ depolarization, Ca^2+^ release and contractile apparatus analyses

Each skinned fiber was initially equilibrated in the K‐HDTA solution for 2 min before depol. The fiber was depolarized by replacing the K‐HDTA with the Na‐HDTA solution for ~5–10 sec, which typically results in Ca^2+^ release and substantial force response, and was then returned to the K‐HDTA solution for 2 min. This procedure was repeated until the peak force responses were stable. Thereafter, the fiber was equilibrated for 1 min in the K‐HDTA solution with 0.5 mmol L^−1^ EGTA and transferred into the Ca^2+^ release solution to elicit caffeine‐induced force. Finally, the fiber was exposed to the max Ca^2+^ solution. Caffeine‐ and depol‐induced forces were evaluated relative to max Ca^2+^‐activated force (caffeine/max Ca^2+^ ratio and depol/max Ca^2+^ ratio, respectively). Max Ca^2+^‐activated force was normalized to the cross‐sectional area of the fibers (specific max Ca^2+^ force). The cross‐sectional area was modeled as a circular profile if the diameter was similar along the fiber segment and was calculated from an average of three width measurements.

The force–[Ca^2+^] relationship was determined by exposing the skinned fiber to a sequence of [Ca^2+^] solutions (10^−9.0^, 10^−6.4^, 10^−6.2^, 10^−6.0^, 10^−5.8^, 10^−5.6^, 10^−5.4^, and ~10^−4.7^ mol L^−1^). The force response produced at each [Ca^2+^] was expressed as a percentage of the maximum force. Using SigmaPlot 12 software (HULINKS, Tokyo, Japan), [Ca^2+^]_50_, which is the [Ca^2+^] at the half‐maximum force, and Hill coefficient (the steepness of the force–[Ca^2+^] relationship) were calculated.

### Experiment 3

#### Experimental protocol

The purpose of the experiment 3 was to examine whether in vitro *S*‐nitrosylation of calpain decreases Ca^2+^‐elicited proteolysis. To this end, in vitro experiments were conducted with rested TA muscles from 8‐ to 9‐week‐old rats (*n* = 5), in which the muscle proteins were first *S*‐nitrosylated and then exposed to high [Ca^2+^].

Tibialis anterior muscles (~100 mg) were homogenized in 5 vol. (vol. mass^−1^) of an ice‐cold buffer composed of 20 mmol L^−1^ Tris/HCl (pH 7.4), 5 mmol L^−1^ EGTA, 0.5 mmol L^−1^ phenylmethanesulfonyl fluoride, 14 *μ*mol L^−1^ pepstatin A, and 10 *μ*g mL^−1^ 4‐(2‐aminoethyl)‐benzenesulfonylfluoride. The homogenate was centrifuged at 200* g* for 10 min at 4°C and the resultant supernatant was collected. To *S*‐nitrosylate the protein thiols, the supernatant was preincubated with 1 mmol L^−1^ *S*‐nitrosoglutathione (GSNO) for 15 min at 37°C. It has been reported previously that when GSNO is applied to proteins immediately after being dissolved in solution, it causes *S*‐glutathionylation but not *S*‐nitrosylation (Dutka et al. 2011b). Therefore, stocked GSNO was always utilized. Thereafter, to activate calpain, CaCl_2_ was added to the sample to produce free [Ca^2+^] of 0.1 mmol L^−1^. After incubation for 15 min at 37°C, calpains were inactivated by adding 10 mmol L^−1^ EDTA and 10 mmol L^−1^ EGTA.

#### Immunoblot and SNO‐RAC

Immunoblot was performed in a manner similar to those used in the experiment 1. To check the assay specificity for *S*‐nitrosylated thiol detection and ascertain whether GSNO treatment can result in *S*‐nitrosylation of calpain‐1, SNO‐RAC was performed in the presence and absence of GSNO and Cu‐Asc treatments (Fig. 8A).

### Statistics

Data are presented as means ± SEM. In experiments 1 and 2, except for force production of skinned fibers, the effects of ECC (rested vs. ECC muscles) and ARG ingestion (CON vs. ARG rats) were investigated using two‐way ANOVA. Data on depol/max Ca^2+^ ratio of skinned fibers, which did not have equal variances, were analyzed using Kruskal–Wallis ANOVA on ranks. In experiment 3, the effects of GSNO and Ca^2+^ were investigated using one‐way ANOVA for repeated measures. When significant differences were detected, Holm–Sidak or the Dunn's methods were used, as appropriate. The acceptable level of significance was set at *P *<* *0.05. Statistical analyses were performed using SigmaPlot version 12.

## Results

### Experiment 1

#### Isometric force output

**Figure 1:**
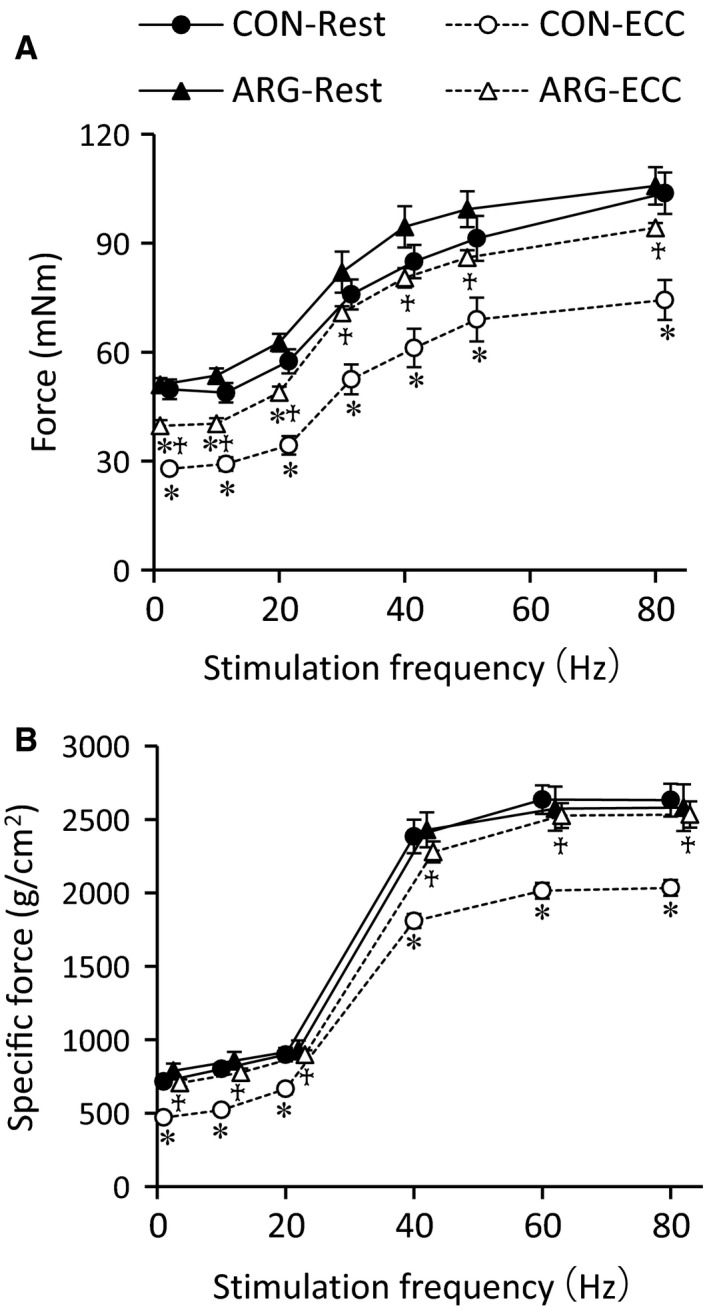
ARG ingestion facilitates restoration of force production after ECC. ARG rats were provided water that contained ARG for 7 days starting from 3 days before the ECC protocol. ECCs were repeated in the anterior muscles of the left hind limb for 200 cycles. The rested muscles of the contralateral (right) legs were used as controls. Three days after ECC, isometric force was measured in situ (A) and in vitro (B). (A) force–frequency relationship in the anterior muscles. Force production was expressed in milli‐Newton‐meters (mNm). (B) specific force–frequency relationship in extensor digitorum longus muscles. Values are means ± SEM (*n* = 7 for each muscle). *P <* *0.05, versus rested muscles within rats; ^†^ *P *<* *0.05, versus ECC muscles from CON rats. ARG, l‐arginine; CON, control; ECC, eccentric contraction.

In a preliminary experiment, we observed that immediately after ECC, force output developed by anterior (TA and EDL) muscles was significantly decreased (by ~50–70%) in both CON and ARG rats and there were no differences in the extent of decreases between them. Three days following ECC, the force in the anterior muscles from CON rats remained low at all stimulation frequencies investigated (Fig. 1A). A greater loss of force was found at low (44% decrease at 1 Hz) than at higher frequencies (28% decrease at 80 Hz). ECC‐related decreases in forces in ARG rats (by ~10–20%) were smaller than those in CON rats; forces at 30–80 Hz did not differ significantly between rested and ECC muscles. The force responses in EDL muscles resembled those in anterior muscles (Fig. 1B). However, the effect of ARG ingestion was more pronounced. Only in CON, but not in ARG rats, significant differences were observed between rested and ECC muscles. These results indicate that ARG ingestion is effective in facilitating restoration of force in TA and EDL muscles after ECC.

#### Contents of Ca^2+^ regulatory proteins

**Figure 2:**
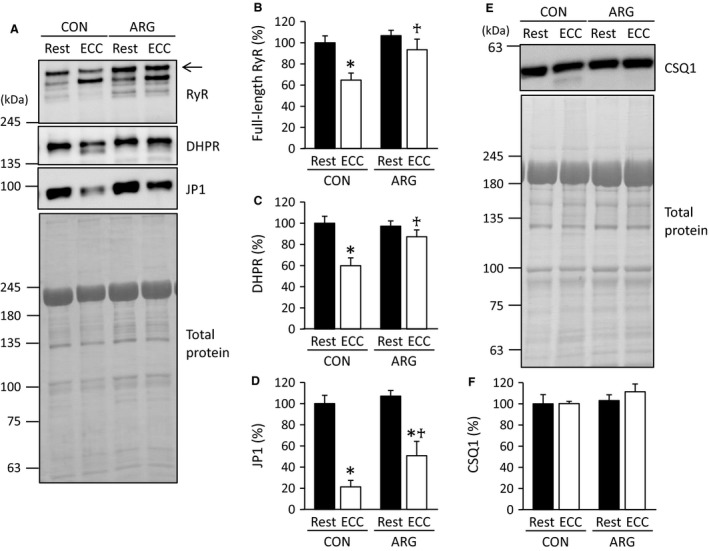
ARG ingestion inhibits ECC‐induced proteolysis of Ca^2+^ regulatory proteins. For the protocols of ARG ingestion and ECC, see legend of Figure 1. (A and E) immunoblot analyses of RyR, DHPR, JP1, and CSQ1 in tibialis anterior muscles. RyR, DHPR, and JP1 were analyzed on the same membrane. For RyR and DHPR, in addition to full‐length proteins, faster migrating bands were observed and most likely corresponded to degraded proteins. Total proteins on the membrane, which were used as a loading control, were visualized with Coomassie blue R staining. Positions of molecular mass makers are indicated on the left. (B, C, D, and F) means ± SEM (*n* = 7 for each muscle) of the contents of full‐length RyR (arrow), DHPR, JP1, and CSQ1, respectively. The contents of four proteins investigated were evaluated relative to total proteins. The results are expressed as percentages of the values observed in rested muscles from CON rats. *P <* *0.05, versus rested muscles within rats; ^†^ *P *<* *0.05, versus ECC muscles from CON rats. ARG, l‐arginine; CON, control; CSQ1, calsequestrin‐1; DHPR, dihydropyridine receptor; ECC, eccentric contraction; JP1, junctophilin‐1; RyR, ryanodine receptor.

Typical samples of immunoblot for RyR, DHPR, JP1, and CSQ1 are shown in Figure 2A and E. For RyR and DHPR, clear bands that migrated faster than full‐length proteins were observed and most likely correspond to degraded proteins (Kanzaki et al. 2017). In CON rats, ECC caused pronounced decreases in the RyR, DHPR, and JP1 contents, which amounted to 65%, 60%, and 21% of those in rested TA muscles, respectively (Fig. 2B–D). It has widely been accepted that decreases in these proteins are ascribable to increased proteolysis (Zhang et al. 2008; Kanzaki et al. 2014, 2017). ARG ingestion resulted in complete or partial inhibition of ECC‐elicited proteolysis. In ARG rats, there were no significant differences in the RyR and DHPR contents between rested and ECC muscles (Fig. 2B and C). For JP1, the contents in ECC muscles decreased to 51% of those in rested muscles, but were 143% higher than those in ECC muscles from CON rats (Fig. 2D). For CSQ1, ECC and/or ARG ingestion had no impact (Fig. 2F).

#### Contents of autolyzed calpain‐1

**Figure 3:**
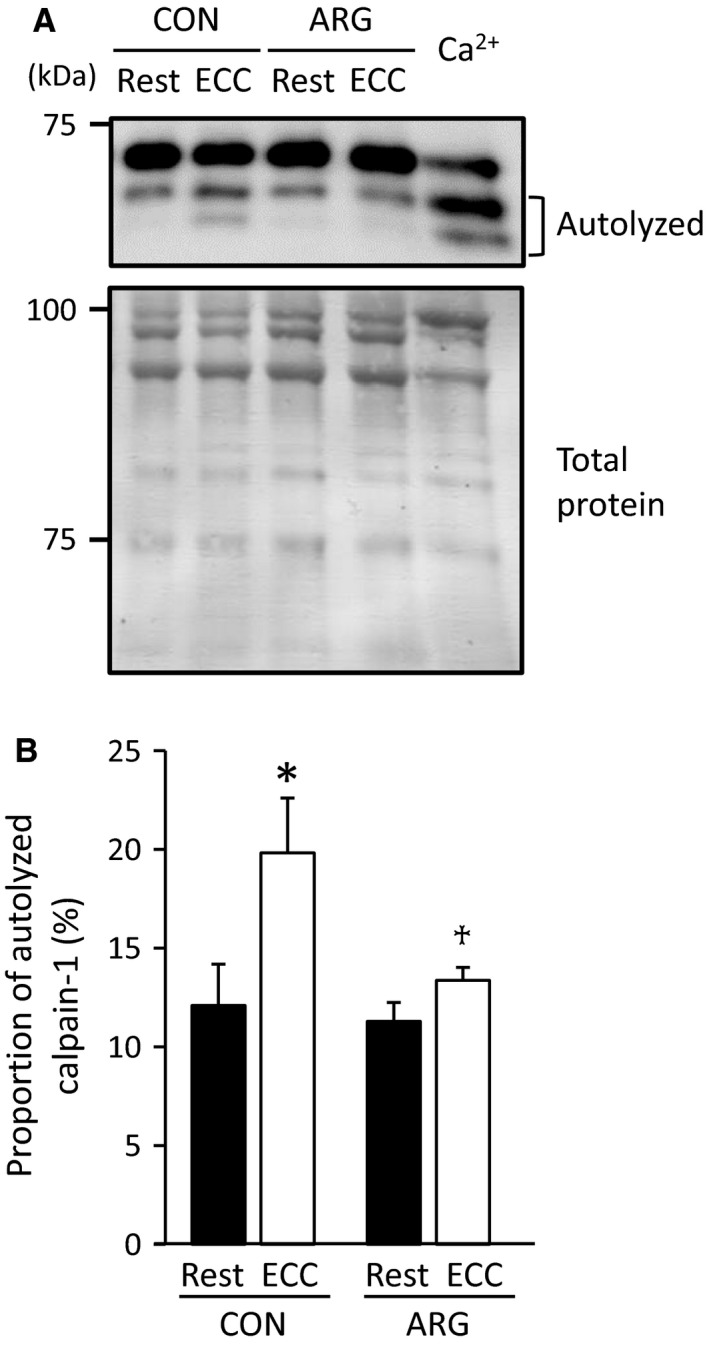
ARG ingestion inhibits ECC‐induced increases in autolyzed calpain‐1. For the protocols of ARG ingestion and ECC, see legend of Figure 1. (A) immunoblot analyses of calpain‐1 in tibialis anterior muscles. A maximum of three bands were discerned. Of these, the upper and the lower two bands represent full‐length and autolyzed forms of calpain‐1, respectively. Standard muscle sample (Ca^2+^) was incubated with 0.5 mmol L^−1^ free [Ca^2+^]. Positions of molecular mass markers are indicated on the left. (B) means ± SEM (*n* = 7 for each muscle) of the proportion of autolyzed calpain‐1. The content of autolyzed form is expressed as a percentage of total calpain‐1. *P <* *0.05, versus rested muscles within rats. ^†^ *P *<* *0.05, versus ECC muscles from CON rats. ARG, l‐arginine; CON, control; ECC, eccentric contraction.

With the consideration that calpain‐1 plays a central role in ECC‐induced proteolysis of Ca^2+^ regulatory proteins (Kanzaki et al. 2014, 2017), we examined the contents of autolyzed calpain‐1, an indicator of its activation. In accordance with previous observations (Murphy et al. 2007; Kanzaki et al. 2014), small amounts (~12–13%) of calpain‐1 were present in autolyzed forms in rested TA muscles (Fig. 3). In CON rats, the contents of autolyzed calpain‐1 were augmented in ECC muscles to 164% of those in rested muscles, whereas they were unaltered in ARG rats.

#### Contents of NOx and *S*‐nitrosylated calpain‐1

**Figure 4:**
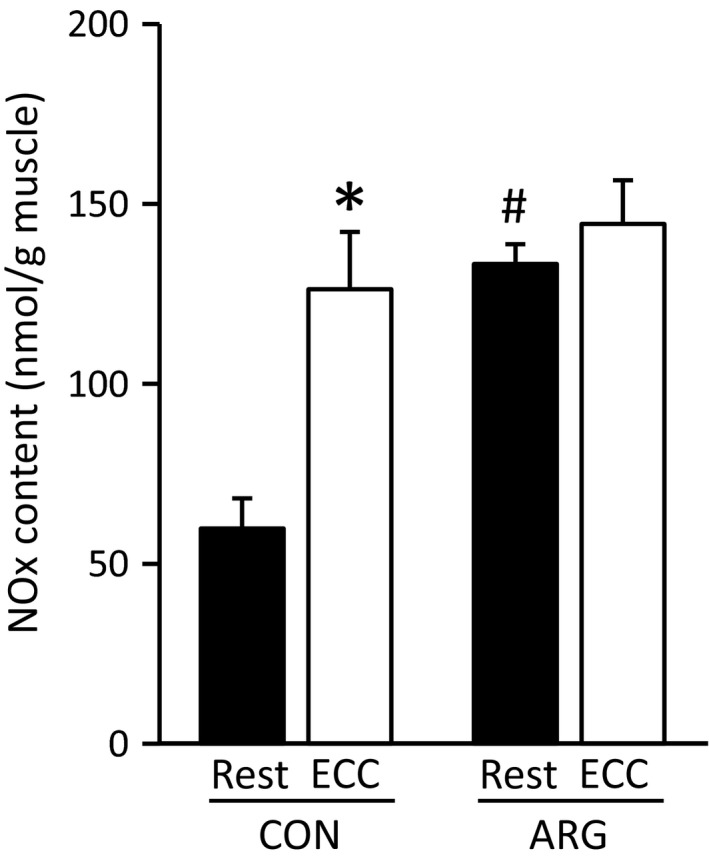
ECC and ARG ingestion increase NOx contents. For the protocols of ARG ingestion and ECC, see legend of Figure 1. The NOx contents in tibialis anterior muscles were spectrophotometrically measured using the Griess reagent. Values are means ± SEM (*n* = 7 for each muscle). *P <* *0.05, versus rested muscles within rats, ^#^ *P *<* *0.05, versus rested muscles from CON rats. ARG, l‐arginine; CON, control; ECC, eccentric contraction; NOx, nitrite and nitrate.

Both ARG ingestion and ECC resulted in pronounced increases in the NOx content (Fig. 4). In CON rats, the NOx content was increased in ECC muscles to 211% of that in rested muscles. In rested muscles from ARG rats, the increases approximately corresponded to those in ECC muscles from CON rats. Interestingly, the additive effects of ARG ingestion and ECC were not observed in ECC muscles from ARG rats; there were no differences between the rested and ECC muscles. These results indicate that ARG and/or ECC treatments cause the increased contents of endogenously produced NO.

**Figure 5:**
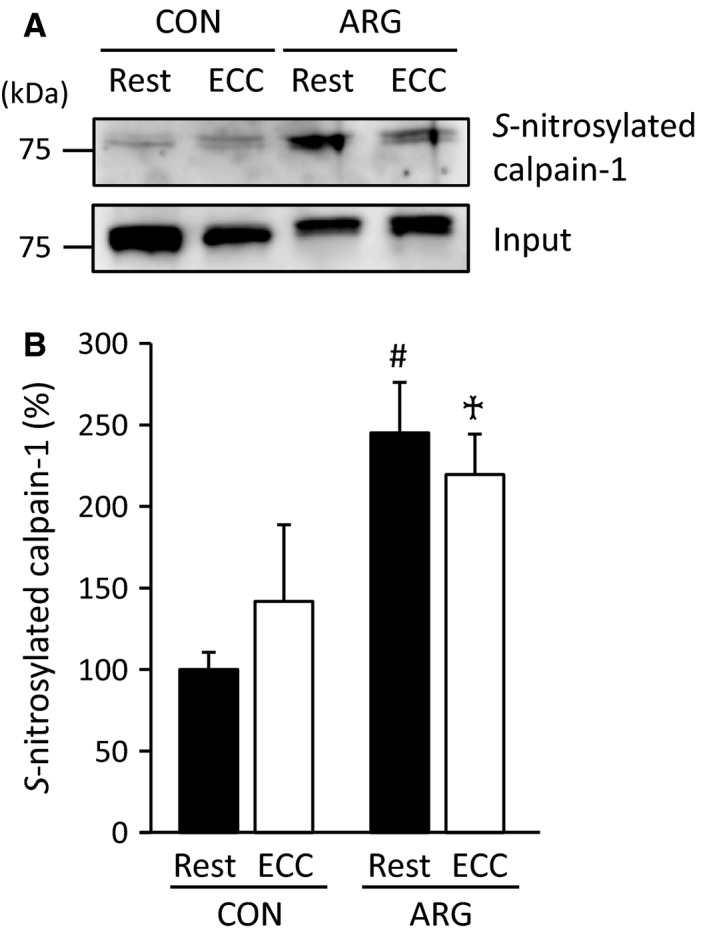
ARG ingestion increases *S*‐nitrosylated calpain‐1. For the protocols of ARG ingestion and ECC, see legend of Figure 1. (A) immunodetection of *S*‐nitrosylated calpain‐1 in tibialis anterior muscles. *S*‐nitrosylated calpain‐1 was detected using the SNO‐RAC technique and immunoblot. The sample (input) before the resin‐assisted capture was used as a loading control. Positions of molecular mass markers are indicated on the left. (B) means ± SEM (*n* = 5 for each muscle) of the contents of *S*‐nitroslyated calpain‐1. The contents of *S*‐nitrosylated calpain‐1 were evaluated relative to the band density of “input”. The results are expressed as percentages of the values in rested muscles from CON rats. ^#^ *P *<* *0.05, versus rested muscles from CON rats, ^†^ *P *<* *0.05, versus ECC muscles from CON rats. ARG, l‐arginine; CON, control; ECC, eccentric contraction; SNO‐RAC, resin‐assisted capture of *S*‐nitrosothiol.

To elucidate whether increased NO can result in *S*‐nitrosylation of calpain‐1, analyses using the SNO‐RAC technique were performed. In CON rats, there were no differences in the contents of *S*‐nitrosylated calpain‐1 between rested and ECC muscles (Fig. 5). On the other hand, in ARG rats, the contents of *S*‐nitrosylated calpain‐1 were increased in both rested and ECC muscles to more than 200% of those in rested muscles from CON rats.

### Experiment 2

#### Myofibrillar Ca^2+^ sensitivity

**Figure 6:**
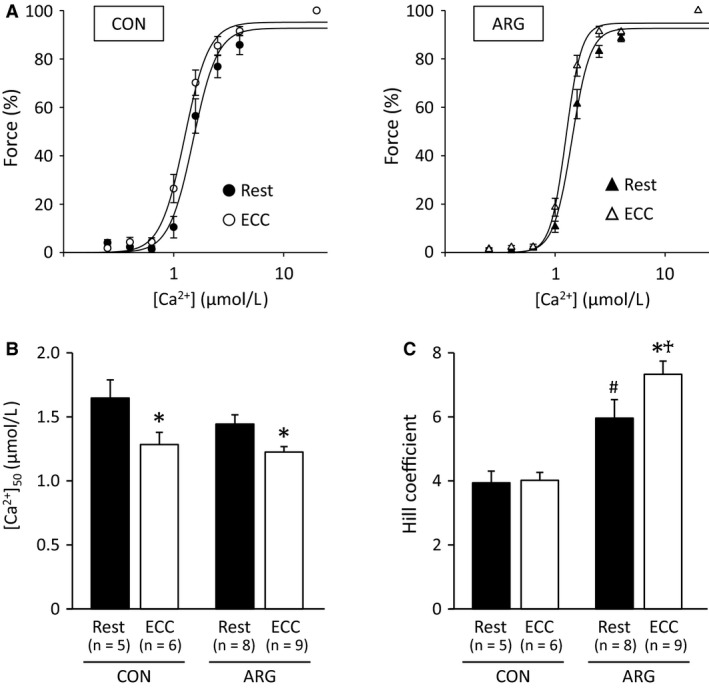
ECC and ARG ingestion increase myofibrillar Ca^2+^ sensitivity. For the protocols of ARG ingestion and ECC, see legend of Figure 1. Mechanically skinned fibers were prepared from tibialis anterior muscles. (A) Hill fits to force‐[Ca^2+^] for CON (left) and ARG (right) rats. Skinned fibers were exposed to solutions at progressively higher free [Ca^2+^] (0.25, 0.40, 0.63, 1.00, 1.56, 2.51, 3.98, and 19.95 *μ*mol L^−1^). (B and C) means ± SEM of [Ca^2+^]_50_ and Hill coefficient, respectively. *P <* *0.05, versus rested fibers within rats, ^#^ *P *<* *0.05, versus rested fibers from CON rats, ^†^ *P *<* *0.05, versus ECC fibers from CON rats. ARG, l‐arginine; [Ca^2+^]_50_, [Ca^2+^] at half‐maximal force; CON, control; ECC, eccentric contraction.

Neither ECC nor ARG ingestion affected specific max Ca^2+^ force in the skinned TA fibers (results not shown), suggesting no changes in the maximal function of the myofibril. A greater loss of force at low stimulation frequencies shown in Figure 1 is widely acknowledged to be related to reduced SR Ca^2+^ release and/or decreased myofibrillar Ca^2+^ sensitivity (Cheng et al. 2017). It is accordingly reasonable to assume that myofibrillar Ca^2+^ sensitivity would be decreased in fibers undergoing ECC. However, ECC had the opposite effects. As shown in Figure 6B, myofibrillar Ca^2+^ sensitivity, as assessed by [Ca^2+^]_50_, was increased in ECC fibers of both CON and ARG rats. The effect of ARG ingestion alone was not observed. Hill coefficient exhibited changes that differ from those of myofibrillar Ca^2+^ sensitivity (Fig. 6C). As judged by the comparison between rested fibers from CON and ARG rats, ARG ingestion alone evoked increases in the Hill coefficient. ECC resulted in further increases in ARG rats, whereas it exerted no effect in CON rats.

**Figure 7:**
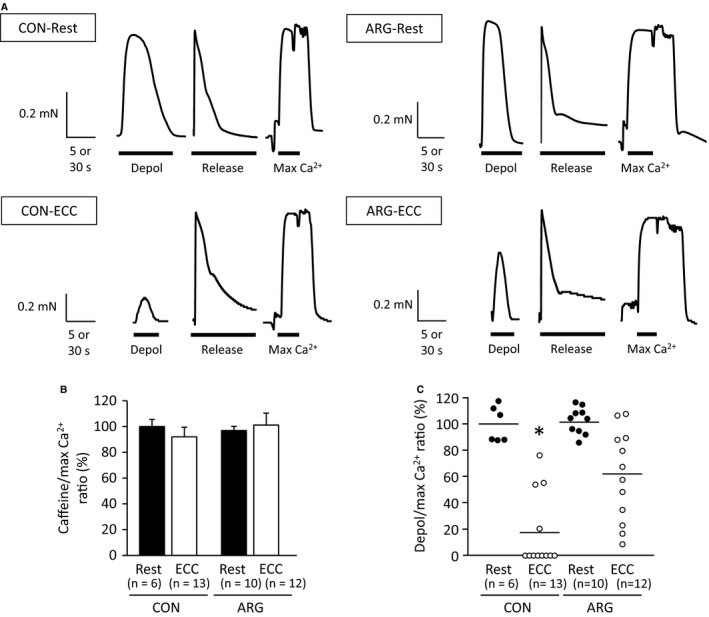
ARG ingestion inhibits ECC‐induced decreases in depolarization‐induced force production. For the protocols of ARG ingestion and ECC, see legend of Figure 1. Mechanically skinned fibers were prepared from tibialis anterior muscles. (A) typical example of force responses to depolarization (depol), Ca^2+^ release, and maximum (max) Ca^2+^ solutions in skinned fibers. Depol‐induced force responses were elicited by replacing the K‐HDTA with the Na‐HDTA solution. (B) means ± SEM of caffeine‐induced force. (C) point blot of depol‐induced force. Caffeine‐ and depol‐induced forces were evaluated relative to the max Ca^2+^‐activated force, and are expressed as percentages of the values in rested muscles from CON rats. Horizontal lines indicate mean values. *P <* *0.05, versus rested fibers within rats. ARG, l‐arginine; CON, control; ECC, eccentric contraction.

#### Caffeine‐ and depol‐induced force

As shown in Figure 7B, there were no significant differences in the caffeine/max Ca^2+^ ratio among the four groups investigated, suggesting that RyR function and Ca^2+^ content contained in the SR were unchanged. Data on the depol/max Ca^2+^ ratio are shown using point blots, in which the individual values for all the fibers are plotted (Fig. 7C), because they did not have equal variances, The “Kruskal–Wallis ANOVA on ranks” test indicated that ARG ingestion had an inhibitory effect on ECC‐related decreases in the depol/max Ca^2+^ ratio; only in CON but not in ARG rats, significant differences in the ratios were observed between rested and ECC fibers, although the data on ECC fibers displayed a pronounced scattering.

### Experiment 3

**Figure 8:**
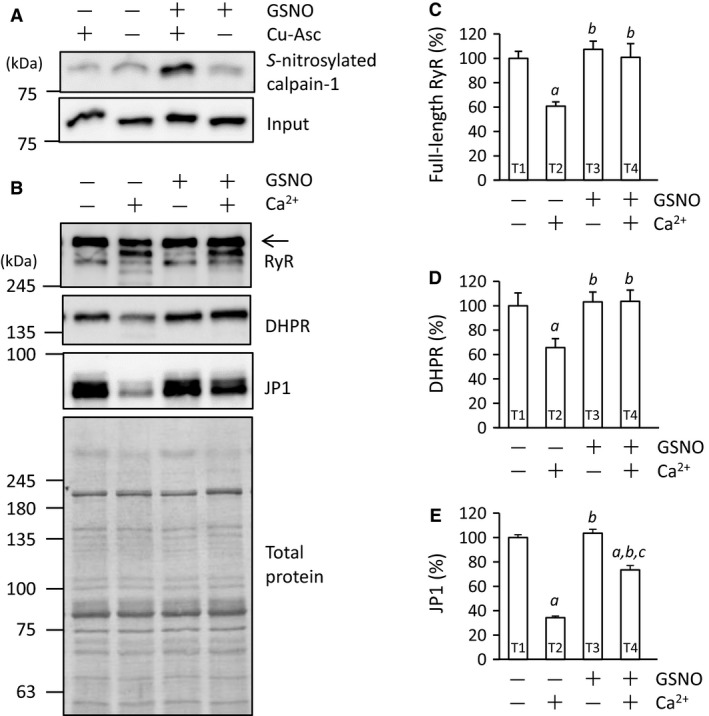
In vitro *S*‐nitrosylation of calpain‐1 inhibits Ca^2+^‐induced proteolysis of Ca^2+^ regulatory proteins. (A) immunodetection of GSNO‐induced *S*‐nitrosylation of calpain‐1 in tibialis anterior muscles. To check the assay specificity and ascertain whether GSNO treatment can result in *S*‐nitrosylation of calpain‐1, SNO‐RAC was performed in the presence (+) and absence (−) of GSNO and Cu‐Asc treatment. Positions of molecular mass markers are indicated on the left. (B) immunoblots of RyR, DHPR, and JP1. Muscle homogenates were incubated in the presence (+) and absence (−) of GSNO and Ca^2+^. Total proteins on the membrane, which were used as a loading control, were visualized with Coomassie blue R staining. (C, D and E) means ± SEM (*n* = 5 for each treatment) of the contents of full‐length RyR (arrow), DHPR, and JP1, respectively. The contents of the three proteins were evaluated relative to the total proteins. The results are expressed as percentages of the values in samples treated without GSNO and Ca^2+^. ^*a*^ *P* < 0.05, versus T1. ^*b*^ *P* < 0.05, versus T2. ^*c*^ *P* < 0.05, versus T3. GSNO,* S*‐nitrosoglutathione; Cu‐Asc, CuCl and sodium ascorbate; DHPR, dihydropyridine receptor; JP1, junctophilin‐1; RyR, ryanodine receptor; SNO‐RAC, resin‐assisted capture of *S*‐nitrosothiols; T, treatment.

As shown in Figure 8A, much larger amounts of calpain‐1 were captured in the presence of both GSNO and Cu‐Asc, compared to three other treatments, indicating the assay specificity for detection of *S*‐nitrosylated proteins and *S‐*nitrosylation of calpain‐1 with 1 mmol L^−1^ GSNO treatment.

In experiment 3, the effects of GSNO and Ca^2+^ on proteolysis were assessed under four conditions, that is, (1) treatment (T) without GSNO and Ca^2+^; (2) T without GSNO and with Ca^2+^; (3) T with GSNO and without Ca^2+^; and (4) T with GSNO and Ca^2+^, which are referred to as T1, T2, T3, and T4, respectively. T2 decreased the RyR, DHPR, and JP1 contents to 61%, 67%, and 34%, respectively, of the value observed with T1 (Fig. 8C–E). T3 and T4 unchanged the RyR and DHPR contents. For JP1, T4 elicited a significant decrease to 73% of the value with T1, but the level was higher than that with T2 (Fig. 8E). Together, the present results are strongly suggestive that ARG ingestion is capable of inhibiting ECC‐induced proteolysis through *S*‐nitrosylation of calpains resulting in the decreased proteolytic function.

## Discussion

There is a large body of literature showing that ECC‐induced force deficits are mainly ascribable to calpain‐1 activation that leads to proteolysis of key proteins involved in the excitation–contraction coupling (Zhang et al. 2012; Murphy et al. 2013; Kanzaki et al. 2014, 2017). To our knowledge, the present investigation is the first to report that ARG ingestion can affect force production in muscles undergoing ECC through the following processes: (1) ARG ingestion increases NO levels in rested muscles; (2) *S*‐nitrosylation of calpain‐1 that occurs as a consequence of increased NO depresses the proteolytic function of the enzyme; (3) inactivation of calpain‐1 inhibits ECC‐dependent proteolysis of Ca^2+^ regulatory proteins; (4) restoration of SR Ca^2+^ release function and force production is facilitated.

Although exogenously applied NO has been shown to elicit *S*‐nitrosylation of calpain‐1 in vitro, which in turn causes decreases in Ca^2+^‐dependent autolysis of calpain‐1 (Liu et al. 2016), the effects of ARG ingestion on *S*‐nitrosylation are yet to be determined. This study revealed that ARG ingestion was able to evoke *S*‐nitrosylated calpain‐1 in vivo (Fig. 5), resulting in a decrease in ECC‐related autolysis of calpain‐1 (Fig. 3). Calpain‐1 is a Cys protease containing 11 and 2 Cys residues in the catalytic and the small subunit, respectively (Liu et al. 2016). An in vitro study using GSNO, a NO donor, indicated that, of these, NO can *S*‐nitrosylate Cys 49, 351, 384, and 592 on the catalytic subunit, and Cys 142 on the small subunit (Liu et al. 2016). It is unclear, however, whether ARG‐related *S*‐nitrosylation occurs in vivo at these five positions, as the inhibitory effect of GSNO is dose dependent (Liu et al. 2016).

The present results regarding NOx agree with previous findings that NO production is accelerated by ECC (Chiang et al. 2009; Sakurai et al. 2013). Intriguingly, we found that despite the elevated NO content, significant increases in *S*‐nitrosylated calpain‐1 were undetectable in ECC muscles from CON rats (Fig. 5). One possible reason for this is that the sources and reactions of NO differ between normal and ARG spared muscles. Damage of the plasma membrane and subsequent infiltration of neutrophil into the muscle cells are among the relatively earlier changes after ECC (Fielding et al. 1993). In the cell containing neutrophil, levels of both NO and superoxide would be elevated as neutrophil is comprised of inducible NOS and NADPH oxidase that releases superoxide. It might be expected that under such conditions, many of NO produced by inducible NOS would interact with superoxide to form peroxynitrite, owing to which calpain‐1 is less *S*‐nitrosylated, and that when NO levels are elevated before ECC, the plasma membrane would be protected from ECC‐induced damage. In support of this assumption, it has been demonstrated that: (1) except for some pathological conditions, peroxynitrate mainly causes nitration rather than *S*‐nitrosylation (Wiseman and Thurmond 2012; Begara‐Morales et al. 2015); (2) the amounts of protein nitration are increased a few days after ECC (Chiang et al. 2009); (3) in rat hearts, increased NOS mediated via cardioselective gene transfer results in reduced damage to the plasma membrane because of ischemia–reperfusion (Szelid et al. 2010).

One of features of ECC‐elicited changes in force is a greater loss of force at low‐ compared to high‐stimulation frequencies (Balnave and Allen 1995; Raastad et al. 2010; Kanzaki et al. 2014). The present results shown in Figure 1A are in accordance with the well‐accepted view. Diminished submaximal force is purported to be due to decreased myofibrillar Ca^2+^ sensitivity and/or reduced SR Ca^2+^ release (Bruton et al. 2008; Watanabe and Wada 2016; Cheng et al. 2017). In this study, the effects of ECC and/or ARG ingestion on these two factors were investigated using mechanically skinned fibers with functional excitation–contraction coupling. Our results on the depol and caffeine/max Ca^2+^ ratios suggest that ECC‐elicited force deficits may stem, at least in part, from disturbances in SR Ca^2+^ release; the depol/max ratio is an indicator of SR Ca^2+^ release (Watanabe and Wada 2016), and that the site that causes impaired Ca^2+^ release may be located in the transverse‐tubule; as judged by the results of the caffeine/max Ca^2+^ ratio, the function of RyR itself is unchanged. These findings are fully consistent with the conclusion drawn by Ingalls et al. (1998).

The fact that Ca^2+^ regulatory proteins have different susceptibilities to ECC‐induced proteolysis is of interest regarding the role of each protein in SR Ca^2+^ release and force production. It has previously been suggested that proteolysis of JP, which tethers the transverse tubule membrane to the SR membrane, contributes to the force deficits in a range of circumstances, including ECC (Corona et al. 2010; Murphy et al. 2013). On the other hand, we observed that in ECC muscles from ARG rats, restoration of force production occurred in conjunction with pronounced decreases in JP1 and no changes in RyR and DHPR (Figs. 1, 2), which seriously challenges the earlier suggestion. These results, together with the finding that the RyR function is unaltered (see above), make it reasonable to assume that DHPR would play a critical role in the regulation of SR Ca^2+^ release.

As reported previously, elevated levels of NO lead to reduced myofibrillar Ca^2+^ sensitivity due to *S*‐nitrosyla‐tion of troponin I (Spencer and Posterino 2009; Dutka et al. 2017). Therefore, we expected that ECC, which results in increased NO, would reduce Ca^2+^ sensitivity. Surprisingly, ECC resulted in the opposite changes (Fig. 6A). A series of studies by Dutka and colleagues (Dutka et al. 2011b, 2017; Mollica et al. 2012) has revealed that in mammalian fast‐twitch muscles, *S*‐glutathionylation of Cys 134 on troponin I induces a marked increase in Ca^2+^ sensitivity. *S*‐glutathionylation is produced either via a reaction of reduced glutathione (GSH) and oxidized Cys residues or via generation of GSH‐derived species. With the consideration that GSH levels in the myoplasm are increased a few days following ECC (Kanzaki et al., unpublished data), it is possible that Cys 134 on fast troponin I might be *S*‐glutathionylated in muscles subjected to ECC.

In this study, we show that ARG ingestion inhibits ECC‐induced proteolysis of Ca^2+^ regulatory proteins and force deficit, which is mediated through decreases in the proteolytic function of calpains due to *S*‐nitrosylation of the enzyme. It is well known that proper physical activity attenuates chronic diseases such as cardiovascular diseases, hypertension, diabetes, and obesity. Acute or unaccustomed exercise is prone to result in long‐lasting force deficits, since muscle activities more or less include an eccentric component. Given that the majority of locomotion in normal daily activities involves submaximal contractions, particularly, force depressions at low‐stimulation frequencies, which are caused by an impaired function of Ca^2+^ regulatory proteins, may contribute to the sensation of muscle weakness. These phenomena will reduce the motivation of people trying to exercise. The present findings suggest that a supplemental ingestion of ARG exerts beneficial effects, such as the restoration of muscle performance after physical activities.

## Conflict of Interest

The authors declare that they have no competing interests.
